# 357. Risk Factors and Antifungal Susceptibility Pattern of *Candida parapsilosis* Candidemia: A Single centre Observation study from Central India.

**DOI:** 10.1093/ofid/ofac492.435

**Published:** 2022-12-15

**Authors:** Rahul Garg, Archana B Wankhade, Padma Das

**Affiliations:** AIIMS Raipur, Raipur, Chhattisgarh, India; AIIMS RAIPUR, Raipur, Chhattisgarh, India; AIIMS Raipur, Raipur, Chhattisgarh, India

## Abstract

**Background:**

*Candida parapsilosis* infection has recently emerged as an important antifungal-resistant nosocomial pathogen having the unique ability to grow on inanimate objects and surfaces. The aim of this study was to analyze the predisposing conditions, outcome, and antifungal susceptibility pattern of candidemia due to *C. parapsilsosis complex.*

**Methods:**

A single-center retrospective observational study from January 2019 to December 2021 of all cases of candidemia was carried out at an 890-bedded University Hospital in central India. Data regarding demographic characteristics and clinical risk factors were collected from the patient’s medical records. Antifungal susceptibility testing was performed; MIC results were interpreted according to CLSI breakpoints (M27-A3). Risk factors and outcome association at the species level were analyzed by using Fisher’s exact test.

**Results:**

Of 211 patients diagnosed with Candidemia during the study period, 53 (25.1%) were infected with C. *parapsilosis* which represented the second most frequently isolated yeast after C. albicans (n=98; 46.4%). A total of 26 (49%) C. parapsilosis isolates were non-susceptible to fluconazole (NSF), which included resistant (n = 20) and susceptible dose-dependent (n = 06) isolates. The median patient age was 63 years.15.3% were neonates. The majority of patients (90%) suffered from multiple comorbidities, diabetes mellitus (43%) being the commonest. A total of 55 % of patients underwent surgical intervention within 30 days from the onset of candidemia. Univariate logistic regression revealed that ICU admission (odds ratio [OR] 2.45), central venous catheter use (OR 2.46), renal impairment (OR 1.687) were more common among FNS isolates than fluconazole-susceptible (FS) isolates (all P < 0.05). The overall crude mortality at 30 days was 36%; higher in patients infected with FNS isolates than FS isolates.

**Conclusion:**

There is an increase in the absolute number of invasive infections by *C. parapsilosis* observed over the past 2 years. At this moment, the percentage of fluconazole non-susceptible *C. parapsilosis* is very high and poses a threat to infected patients and has a clinical impact in our hospital. Being able to identify and treat infections caused by this pathogen is important to prevent clinical outbreaks.

**Disclosures:**

**All Authors**: No reported disclosures.

